# JMM Profile: Carbapenems: a broad-spectrum antibiotic

**DOI:** 10.1099/jmm.0.001462

**Published:** 2021-12-10

**Authors:** Tom Armstrong, Samuel Jacob Fenn, Kim R. Hardie

**Affiliations:** ^1^​ School of Chemistry, Biodiscovery Institute, University Park, Nottingham University, Nottingham, NG7 2RD, UK; ^2^​ School of Life Sciences and National Biofilm Innovation Centre, Biodiscovery Institute, University Park, Nottingham University, Nottingham, NG7 2RD, UK

**Keywords:** antimicrobial resistance, β-lactams, last resort

## Abstract

Carbapenems are potent members of the β-lactam family that inhibit bacterial cell-wall biosynthesis inhibitors . They are highly effective against Gram-negative and Gram-positive drug-resistant infections . As such, carbapenems are typically reserved as an antibiotic of last resort. The WHO lists meropenem as an essential medicine. Nausea and vomiting are reported in ≤20% of carbapenem recipients, with 1.5% suffering seizures. Enzymatic hydrolysis of the β**-**lactam ring is the main driver of clinical resistance. These enzymes can be classified as Class A, B and D. Classes A and D are serine β**-**lactamases, whereas Class B rely on metal-mediated hydrolysis, typically through zinc.

## Historical perspective

Thienamycin, the first reported carbapenem, was isolated from *Streptomyces cattleya* in 1976 [1]. Despite showing significant potency, clinical use was limited by its instability in water. Since then, medicinal chemistry optimization has yielded a number of carbapenems, namely imipenem, meropenem, doripenem, ertapenem, biapenem and tebipenem, which have been approved for clinical use. However, biapenem and tebipenem are approved for use only in Japan. Tebipenem is currently under development in the USA and has completed Phase III clinical trials.

## Structure/chemistry

**Fig. 1.**
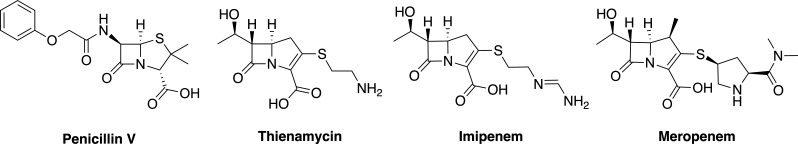
Structure of commonly administered carbapenems in comparison to the β-lactam penicillin V.

Carbapenems are members of the β-lactam class. As shown in Fig. 1, in contrast to penicillins, the 4:5 fused ring system in carbapenems is unsaturated and has no ring sulphur; instead, sulphur is a C2 substituent [2]. The *trans* configuration of C5–C6 and the C-6 (*R*)-hydroxyethyl substituent provides better resistance against β-lactamases. Introducing a methyl group in meropenem at the 1β position increases stability towards the renal dehydropeptidase (DHP-1). In the absence of this methyl group, imipenem must be co-administered with a DHP inhibitor, cilastatin. The thioether component is the main site of modification in carbapenems. Carbapenems bearing substituted pyrrolidine rings (meropenem, ertapenem and doripenem) are particularly potent. Conversion of the carboxylic acid moiety to its corresponding pivoxil ester provides tebipenem with effective oral bioavailability. Biapenem, bearing a pyrazolotriazolium thioether substituent, shows increased stability towards metallo-β-lactamases, which are implicated in carbapenem resistance.

## Mode of action

As with other β-lactam antibiotics, carbapenems are inhibitors of cell-wall biosynthesis; specifically, they prevent transpeptidation, which is integral to the structural integrity of the bacterial cell wall. Inhibition of peptidoglycan crosslinking results in cell lysis and cell death; as such, carbapenems are classified as bactericidal. Broadly speaking, as a compound class, carbapenems show limited permeability of the outer membrane of Gram-negative bacteria. For this reason, carbapenems are reliant on the presence of outer membrane protein (OMP) porins to facilitate their entry into the Gram-negative bacteria; for example, OprD is implicated as a key transport channel for carbapenems, facilitating entry into *Pseudomonas aeruginosa* [3]. Carbapenems inhibit the penicillin-binding-protein (PBP) family of enzymes by acylating the targets. Carbapenem affinity for the different PBPs varies between bacteria, and Gram-negative PBPs are structurally different from those of Gram-positive organisms. A range of PBPs exist and are targeted by different carbapenems; for example, PBP2 is the primary target of meropenem in *Escherichia coli *, whereas high-affinity binding to PBP1, PBP2 and PBP4 is observed in *Staphylococcus aureus *. The basis for this inhibition is the similarity of the β-lactam core to the d-Ala–d-Ala terminus of the peptidoglycan substrate. This structural similarity facilitates covalent binding and thus irreversible inhibition of the target enzymes [4].

**Fig. 2.**
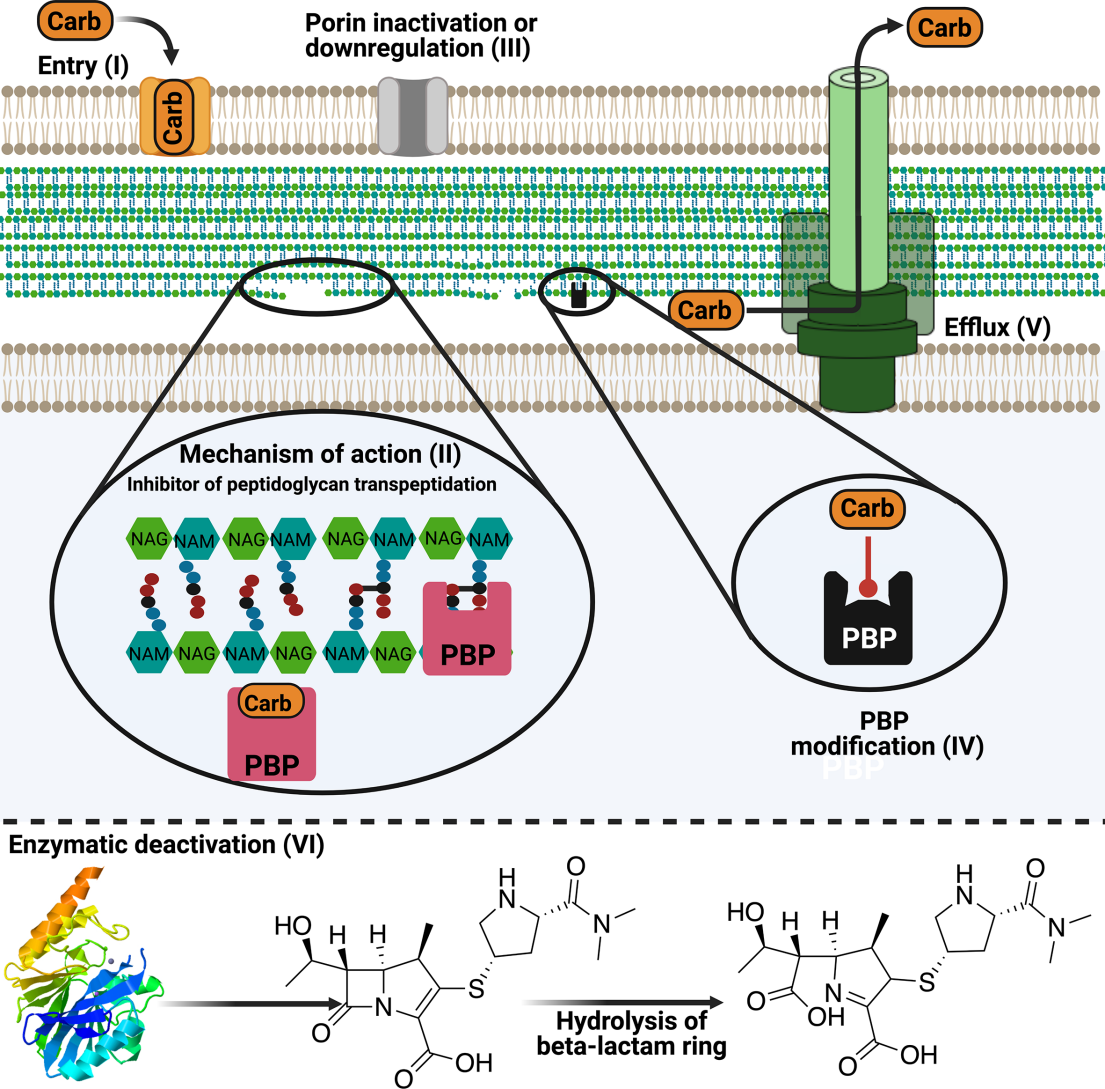
The spectrum and mode of action, plus resistance mechanisms, for carbapenems. Carbapenems enter the bacterial envelope (I) and interact with penicillin binding proteins (PBPs) to disrupt cell-wall biosynthesis leading to cell death (II). Porin downregulation prevents drug molecules from reaching the cellular environment (III). Production of low-affinity PBPs can create resistant strains (IV). Enzymatic hydrolysis of the β-lactam ring deactivates the drug compounds (VI). Upregulation of efflux pumps effectively removes the cytotoxic payload from the cell, reducing (or negating) its therapeutic effects (V).

In terms of metabolism, imipenem is rapidly metabolized by the human kidney enzyme, DHP-1. For this reason, co-administration is performed with cilastatin to increase *in vivo* half-life, to increase tissue penetration and to prevent nephrotoxicity. By contrast, meropenem and ertapenem show greater stability towards the DHP-1 system and can be administered without cilastatin. In the case of meropenem, roughly 70% of the dose is excreted, unchanged in urine over 12 h. Approximately 80% of ertapenem is recovered in urine, with nearly half intact and the other half as the ring-opened metabolite. Due to the significant renal elimination of carbapenems, patients with reduced renal function require adjusted dosages/dosing intervals to avoid drug accumulation (see Fig. 2).

## Mechanisms of microbial resistance

Carbapenem resistance can be both intrinsic and acquired [5]. In Gram-positive bacteria, resistance is primarily mediated by acquiring altered PBPs that are not susceptible to carbapenems. These PBPs appear to be primarily acquired through horizontal transfer from related species. For example, resistance in *Staphylococcus aureus* and *Enterococcus faecium* is associated mainly with variants in PBP2a and PBP5, respectively.

Resistance in Gram-negative bacteria arises through three main mechanisms. (**i**) The first is production of β-lactamase enzymes (carbapenemases) which deactivate the drugs before they elicit their therapeutic effect. β-Lactamases exist as two different structural classes based on their modes of action, serine-dependent (Ambler Class A and D) and metal-dependent (Ambler Class B, also named metallo-β-lactamase). Of particular concern is the acquisition of multiple resistance mechanisms, as seen in *P. aeruginosa* that significantly diminishes carbapenem effectiveness. Gram-negative bacteria can also harbour multiple carbapenemase-encoding genes. The ability to coexpress several carbapenemases is particularly troublesome as a single carbapenem is not sufficient to confer protection. (ii) Overexpression of efflux pumps effectively removes the drugs from the cellular environment, and (iii) porin-mediated resistance restricts entry to the periplasm, which is the primary driver of resistance in *P. aeruginosa *. These factors reinforce the need for responsible antibiotic stewardship [6].

The emergence and spread of carbapenem-resistant *Enterobacteriaceae* (CRE) is a cause for significant concern. Such infections have been observed in a range of bacteria, including *Escherichia coli* and *Klebsiella pneumoniae *. The key driver of resistance in *K. pneumoniae* is the serine-containing carbapenemase *bla*_kpc_ [7]. The gene coding for this enzyme is found on a transposon, which facilitates transmission. CRE can be challenging to identify as some strains harbouring the *bla*_pkc_ gene demonstrate MICs within the acceptable range for carbapenems. The low values mean these strains are not correctly identified as CRE, so appropriate infection controls are not taken. To identify CRE infections, it is recommended that a modified Hodges test (MHT) be used to characterize infection showing elevated carbapenem MICs (see Fig. 2)

## Clinical efficacy and contraindications

### Target organisms

Carbapenems display broad-spectrum activity against Gram-negative bacteria and slightly reduced activity against Gram-positive infections. Meropenem is often used to target serious hospital-acquired infections of unknown aetiology. Imipenem is more active against the Gram-positive cocci, whereas meropenem has higher activity against Gram-negatives.

Doripenem is particularly active against *P. aeruginosa* with lower MICs than imipenem and meropenem. Ertapenem can be used prophylactically for patients undergoing invasive surgery. Meropenem can be used to treat meningitis due to its ability to penetrate meninges. Compared to imipenem and meropenem, ertapenem has a longer half-life and slightly narrow spectrum of use and is ineffective against *P. aeruginosa *. Empiric treatment of unknown β-lactam-resistant infections typically combines a carbapenem and one other drug. Carbapenems do not have activity against *Enterococcus faecium *, Methicillin-resistant *Staphylococcus aureus* (MRSA) or *Stenotrophomonas maltophilia *. Biapenem shows greater activity against *Enterobacteriaceae* than imipenem and two-fold more potency against *P. aeruginosa *. Tebipenem, the first orally available carbapenem, is under development in the USA. It has completed Phase III trials and is active against drug-resistant *Streptococcus pneumoniae* plus a range of *Enterobacteriaceae *; however, its spectrum of activity does not cover MRSA or *P. aeruginosa *. Tebipenem is also used to treat pneumonia in children.

In combination with clavulanic acid, meropenem has also shown activity against *Mycobacterium tuberculosis *, though this is not currently in use as clinical treatment, and further investigations are required.

### Route of delivery

Carbapenems generally have low bioavailability and so must be administered intravenously. Imipenem-cilastatin and ertapenem can also be administered intramuscularly. In addition, tebipenem can be administered orally, though it is only marketed in Japan.

### Dosage (general guidelines)

Generally available as powder or solutions for intravenous administration: meropenem (1 g/30 ml), ertapenem (1 g/20 ml), doripenem (250–500 mg/10 ml) and imipenem with cilastatin sodium at 250–500 mg/20–100 ml.

### Adverse effects/Toxicity

The most common side effects of carbapenem treatment are abdominal pain, nausea, and vomiting, with these symptoms occurring in up to 20% of patients. Swelling and soreness are typically observed at the site of injection. A small number of patients (1.5%) suffer from seizures, but this is generally associated with high-dose treatments. Imipenem presents the highest risk of seizures of all the carbapenems. Nephrotoxicity is associated with imipenem if administered without cilastatin due to the renal metabolism of the carbapenem compound. Some patients have reported phycological changes (anxiety or increased aggression), but the frequency of these side effects is unknown. Carbapenem treatment is avoided in patients who have previously demonstrated allergic reactions to β-lactam antibiotics.

### Contraindications

**Fig. 3.**
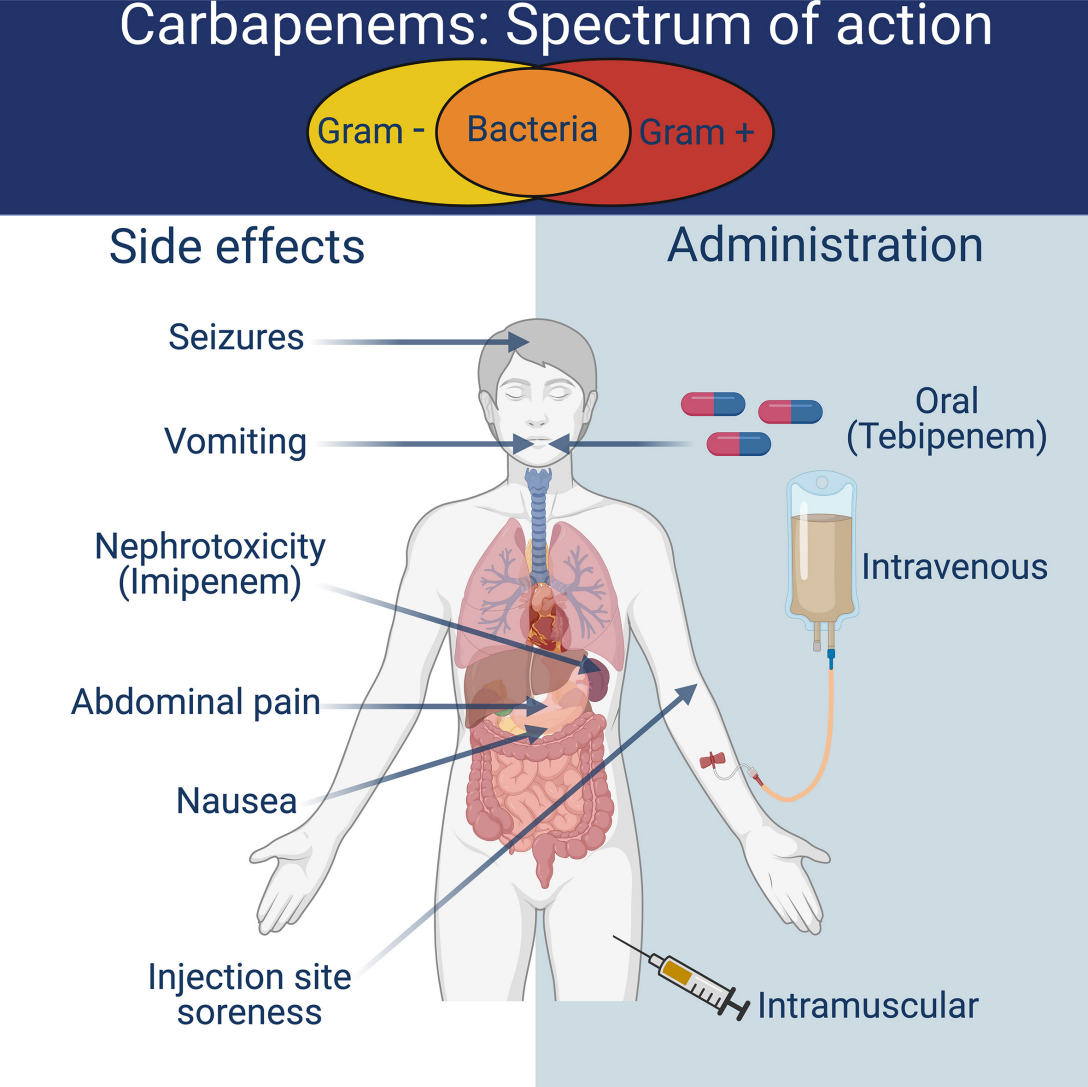
The spectrum of action, side effects and route of delivery for carbapenems.

Carbapenems have been shown to reduce the serum concentration of the anti-seizure drug valproic acid to a sub-therapeutic level. If carbapenem treatment is required, patients should be given an alternative treatment to valproic acid [8]. Imipenem, in particular, has been shown to potentially induce seizures in children with central nervous system infections or people with brain lesions or epilepsy [9]. The propensity of carbapenems to cause seizures is linked to their ability to bind to γ-aminobutyric acid (GABA) receptors, and individuals with a history of seizures are of particular risk. Carbapenem usage is also associated with increased incidence of *Clostridium difficile *-associated diarrhoea in surgical patients. Imipenem has been linked to bone marrow suppression via IgG or IgM antibodies binding to antimicrobial antigens on cell surfaces. This suppression results in thrombocytopenia following lysis or phagocytosis of platelet cells, so caution should be taken in patients with already suppressed platelet counts. Given that the primary route of elimination for carbapenems is via renal excretion, individuals with a history of kidney disease must be closely monitored when receiving carbapenem treatment. Dosing must be adjusted in line with the patient’s kidney function to avoid drugs accumulating to a dangerous level. Some allergic cross-hypersensitivity exists between penicillins and carbapenems, so carbapenem use should be avoided in patients who have previously displayed β-lactam allergies (See Fig. 3).

## Open questions

1. What comes after an ‘antibiotic of last resort’?
2. What is the scope for improving the bioavailability of carbapenems to allow oral delivery?
3. Should carbapenems be removed from empiric therapy to protect against the selection of resistance?
4. Should carbapenems be administered alongside carbapenemase inhibitors?
5. Could carbapenems be applied to multi- and extensively drug-resistant *Mycobacterium tuberculosis* infections?
